# Patient with H syndrome, cardiogenic shock, multiorgan infiltration, and digital ischemia

**DOI:** 10.1186/s12969-021-00586-2

**Published:** 2021-06-30

**Authors:** Laura Ventura-Espejo, Inés Gracia-Darder, Silvia Escribá-Bori, Eva Regina Amador-González, Ana Martín-Santiago, Jan Ramakers

**Affiliations:** 1 Paediatric Department, Hospital Universitario Son Espases, Carretera de Valldemosa, 79, 07120 Palma, Spain; 2 Dermatology Department, Hospital Universitario Son Espases, Palma, Spain; 3 Paediatric Radiology Department, Hospital Universitario Son Espases, Palma, Spain; 4 Multidisciplinary Group for Research in Peadiatrics. Hospital Universitari Son Espases, Balearic Islands Health Research Institute (IdISBa), Carretera de Valldemossa, 79, 07120 Palma, Spain

**Keywords:** H syndrome, Cardiogenic shock, Multiorgan infiltration, Digital ischemia, Paediatric intensive care unit, Interleukin-6, Tocilizumab, CT-scan, Case report

## Abstract

**Abstract:**

**Background:**

H syndrome (HS) is a rare autoinflammatory disease caused by a mutation in the solute carrier family 29, member 3 (SCL29A3) gene. It has a variable clinical presentation and little phenotype-genotype correlation. The pathognomonic sign of HS is cutaneous hyperpigmentation located mainly in the inner thighs and often accompanied by other systemic manifestations. Improvement after tocilizumab treatment has been reported in a few patients with HS. We report the first patient with HS who presented cardiogenic shock, multiorgan infiltration, and digital ischemia.

**Case presentation:**

8-year-old boy born to consanguineous parents of Moroccan origin who was admitted to the intensive care unit during the Coronavirus Disease-2019 (COVID-19) pandemic with tachypnoea, tachycardia, and oliguria.

Echocardiography showed dilated cardiomyopathy and severe systolic dysfunction compatible with cardiogenic shock. Additionally, he presented with multiple organ dysfunction syndrome. SARS-CoV-2 polymerase chain reaction (PCR) and antibody detection by chromatographic immunoassay were negative.

A previously ordered gene panel for pre-existing sensorineural hearing loss showed a pathological mutation in the SCL29A3 gene compatible with H syndrome.

Computed tomography scan revealed extensive alveolar infiltrates in the lungs and multiple poor defined hypodense lesions in liver, spleen, and kidneys; adenopathy; and cardiomegaly with left ventricle subendocardial nodules.

Invasive mechanical ventilation, broad antibiotic and antifungal coverage showed no significant response. Therefore, Tocilizumab as compassionate use together with pulsed intravenous methylprednisolone was initiated. Improvement was impressive leading to normalization of inflammation markers, liver and kidney function, and stabilising heart function. Two weeks later, he was discharged and has been clinically well since then on two weekly administration of Tocilizumab.

**Conclusions:**

We report the most severe disease course produced by HS described so far in the literature. Our patient’s manifestations included uncommon, new complications such as acute heart failure with severe systolic dysfunction, multi-organ cell infiltrate, and digital ischemia.

Most of the clinical symptoms of our patient could have been explained by SARS-CoV-2, demonstrating the importance of a detailed differential diagnosis to ensure optimal treatment.

Although the mechanism of autoinflammation of HS remains uncertain, the good response of our patient to Tocilizumab makes a case for the important role of IL-6 in this syndrome and for considering Tocilizumab as a first-line treatment, at least in severely affected patients.

## Background

HS (OMIM #612391) is an autosomal recessive disorder caused by homozygous or compound heterozygous mutation in SLC29A3, the gene on chromosome 10q22 that encodes human equilibrative nucleoside transporter-3 (hENT3) [1].

HS was first described in 2008 in 6 consanguineous Arabic families [2]. Since then, around 100 cases have been reported [3–5]. The average age at onset is 9.7 years [6].

Previous studies have described other diseases caused by mutations in the SLC29A3 gene, such as pigmented hypertrichosis dermatosis with insulin-dependent diabetes syndrome (PHID), Faisalabad histiocytosis, and familial Rosai Dorfman disease, among others. Many patients shared overlapping signs and symptoms, leading to the suggestion that they should be regarded as the same entity [7–10].

The pathognomonic sign of HS is cutaneous hyperpigmentation located mainly in the inner thighs and often accompanied by hypertrichosis and progressive sclerodermatous induration. Other manifestations include histiocytosis, hepatosplenomegaly, heart anomalies, sensorineural hearing loss (SNHL), exophthalmos, endocrinopathy such as insulin-dependent diabetes mellitus (IDDM), genital abnormalities, and fixed flexion contractures of proximal interphalangeal joints [2–4, 7, 11]. Histologically, skin lesions show a perivascular dermal and subcutaneous infiltrate, composed mainly of histiocytes and plasma cells later replaced by fibrosis.

HS shows a high variability in clinical presentation with a lack of phenotype-genotype correlation even in siblings with identical mutations [3, 7, 12].

We expand the clinical spectrum of HS describing the first patient who presented cardiogenic shock and multiorgan cell infiltrate.

## Case presentation

We report an 8-year-old boy born to consanguineous parents of Moroccan origin who was admitted to the intensive care unit because of dyspnoea, fever, and intense abdominal pain during the COVID-19pandemic. His medical history was positive for SNHL, diagnosed at five years of age, and a gene panel had recently been ordered for this reason. A few months previously, he had suffered an episode of Henoch-Schönlein purpura; and during a follow-up visit, a linear indurated patch was observed on the left thigh. His 4-year-old sister had been diagnosed with IDDM seven months before.

**Fig. 1:**
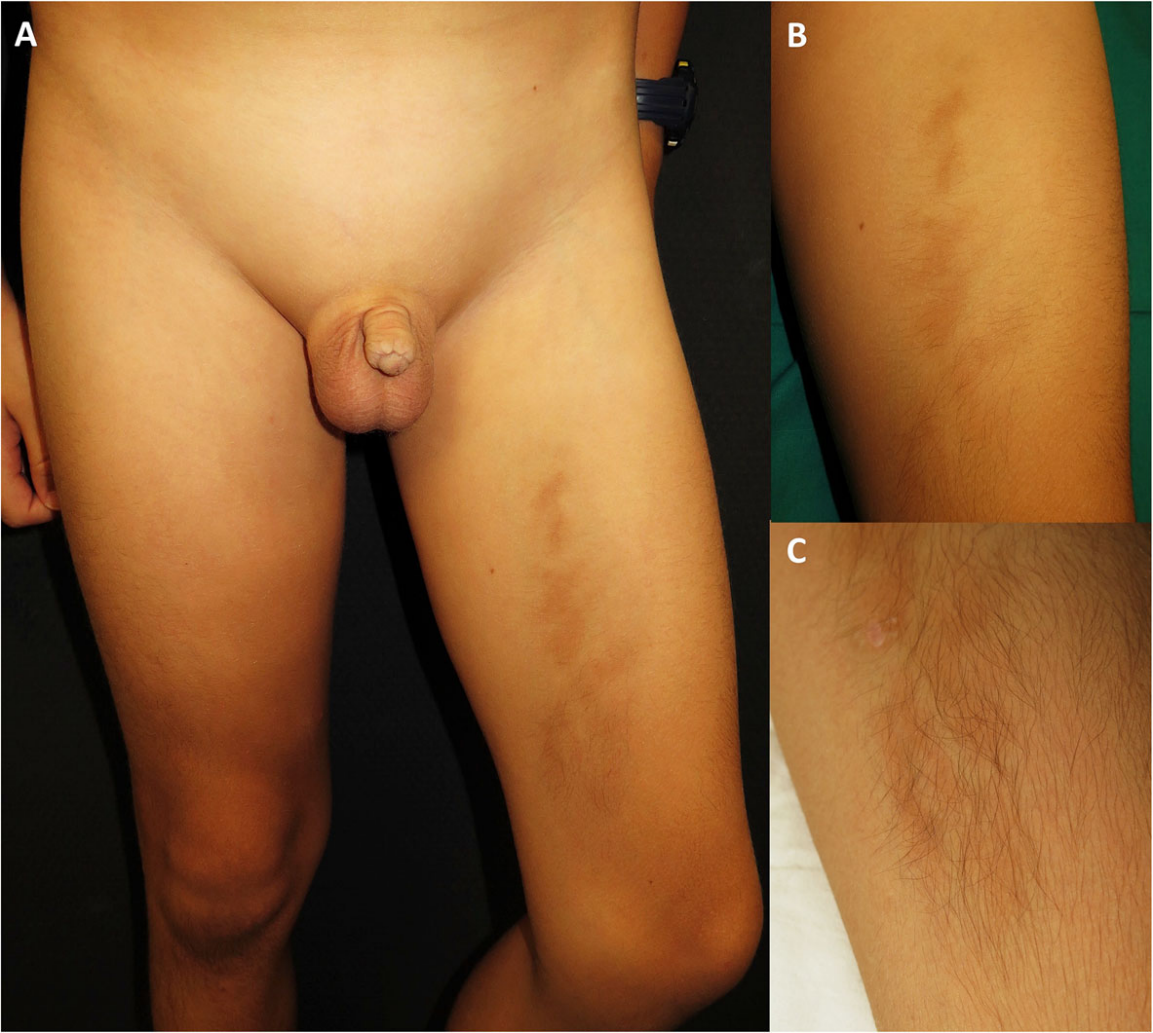
A) Oedema of pubis and scrotum. B) Linear hyperpigmented and indurated plaque with poorly defined edges on the anterior medial aspect of the left thigh. C) Hypertrichosis on the distal region of the hyperpigmented plaque

**Fig. 2:**
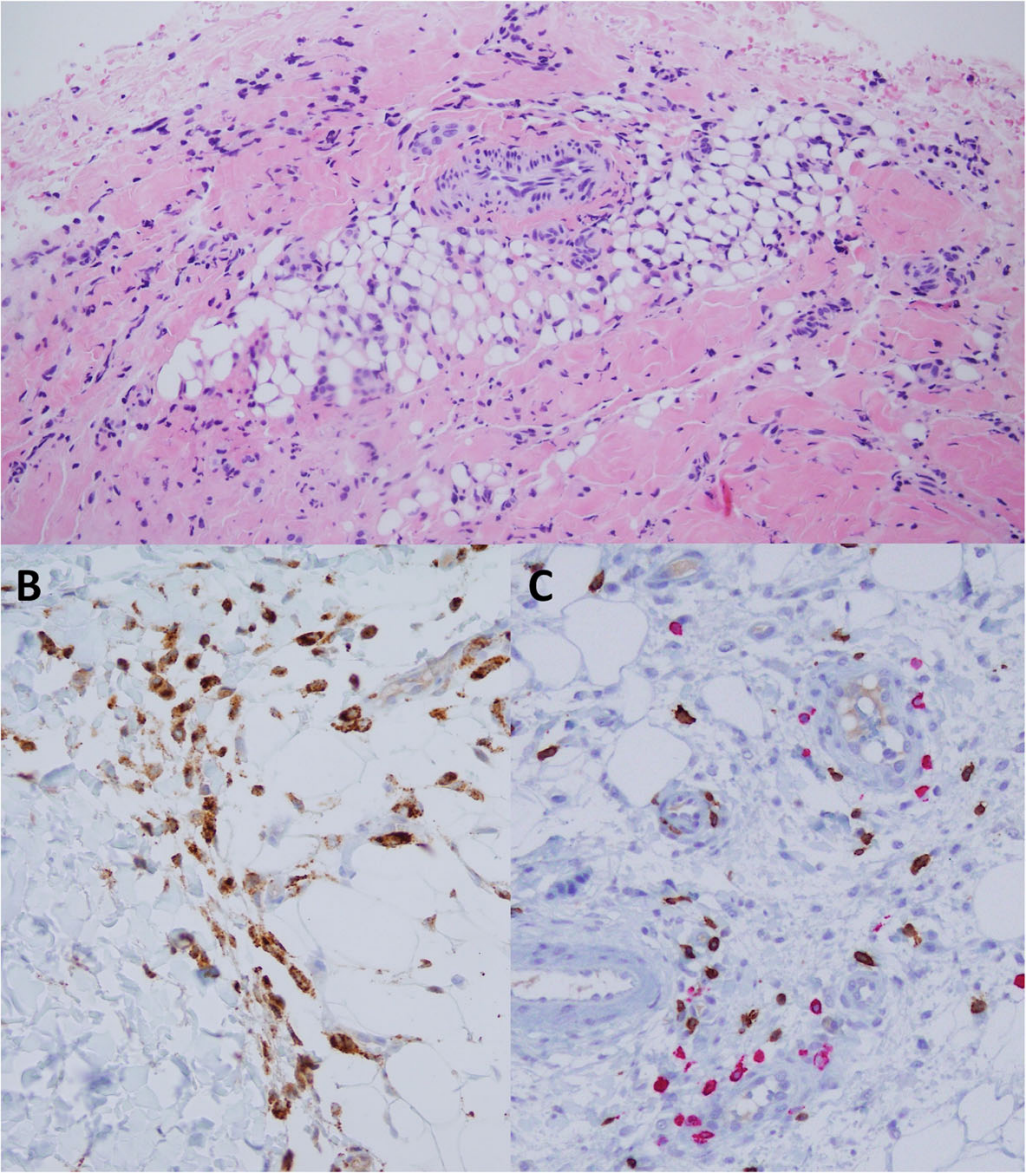
A) Lymphohistiocytic inflammatory infiltrate with areas of lipoatrophy in subcutaneous tissue. B) Presence of histiocytes in the hypodermis (IHQ CD68 60x). C) Infiltrate contains CD3 (brownish) and CD20 (fuchsia) cells in equal proportion, 40x

**Fig. 3:**
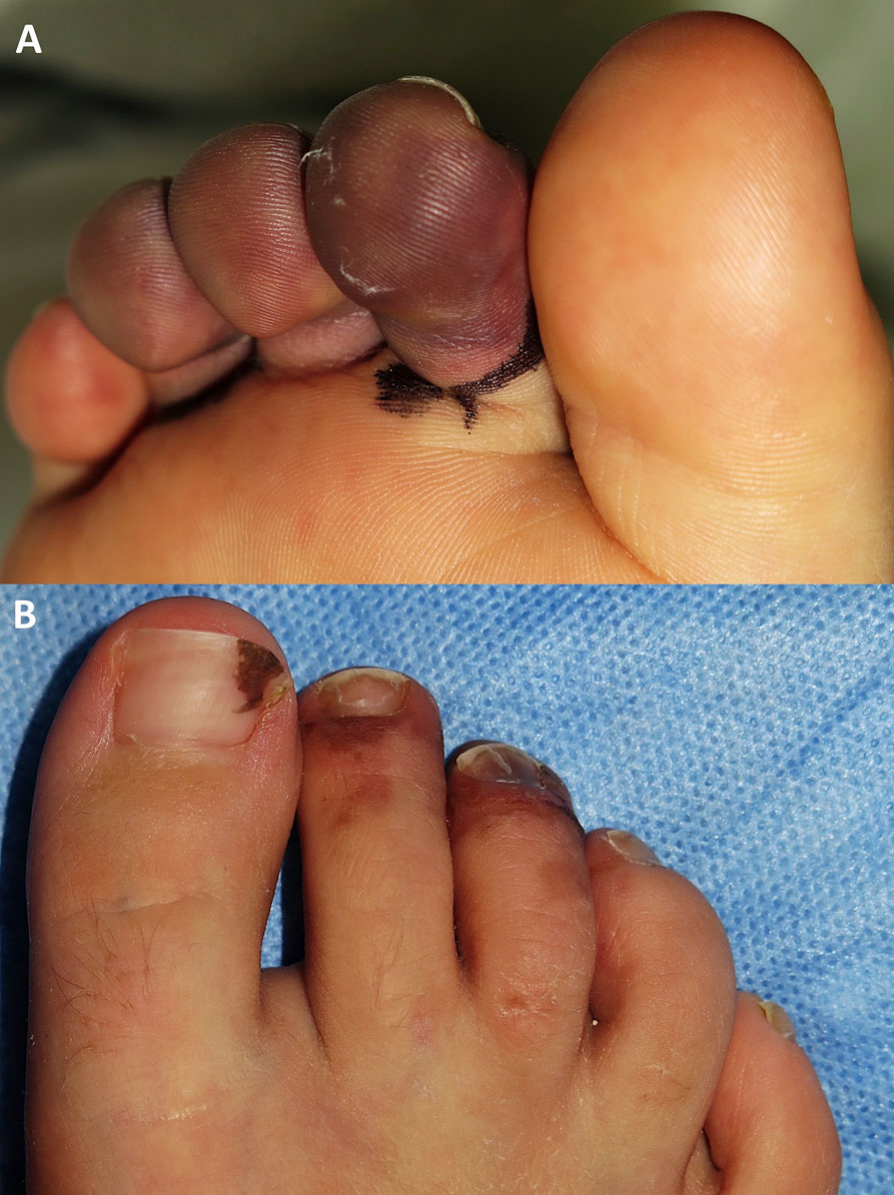
Purpuric macules on the ventral and distal-dorsal side of the 2nd, 3rd, and partially 4th toes

On admission, he showed tachypnoea, tachycardia, and oliguria. Physical exploration showed weak cardiac sounds, gallop rhythms, crackling, hypoventilation, and oedema of scrotum and mons pubis. The skin patch located on the inner left thigh had increased in size and showed thickening, in addition to hypertrichosis and hyperpigmentation (Fig. 1); histologically, the patch exhibited oedema with a lymphohistiocytic infiltrate in subcutaneous and perivascular cell tissue, but with no signs of thrombophlebitis or vasculitis (Fig. 2). Moreover, he developed purpuric lesions of ischemic aetiology in the 2nd and 3rd toes, similar to the lesions described in COVID-19 in children (Fig. 3). The echocardiography detected a dilated cardiomyopathy (left ventricle end- diastolic volume 54 mm Z-score+ 2,9), severe systolic dysfunction (30% of left ventricular ejection fraction (LVEF)), mild diastolic dysfunction (fusion of E/A, E/E’ 16) and elevated pulmonary arterial pressure (tricuspid regurgitation gradient 46 mmHg) confirming cardiogenic shock.

Acute phase reactants were raised, and hepatic and renal function markers were altered (Table 1). High levels of NT-proB-type natriuretic peptide (> 35,000 pg/mL), Renin (4098 μU/mL), and troponin I (59.6 ng/L) were in line with heart failure. Pharyngeal swab test was negative for SARS-CoV-2 and there was no history suggestive of COVID-19 in his family or immediate environment either. Antibody detection by chromatographic immunoassay during the acute phase and after 6 weeks were negative.

**Table 1:**
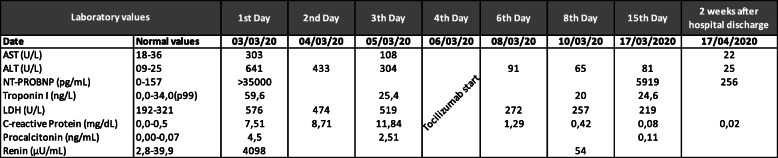
Laboratory values (with normal values) of our patient over time

After detecting a mutation in the SCL29A3 gene (NM_018344.6:c.971C > T; (p.Pro324Leu)), described as pathological [4, 13] in the gene panel ordered for SNHL, the patient was diagnosed with HS, which was compatible with his phenotype. Later on, because early-onset IDDM is a typical feature of HS [3, 6, 12], a genetic panel was done to his sister and the same mutation was found.

**Fig. 4:**
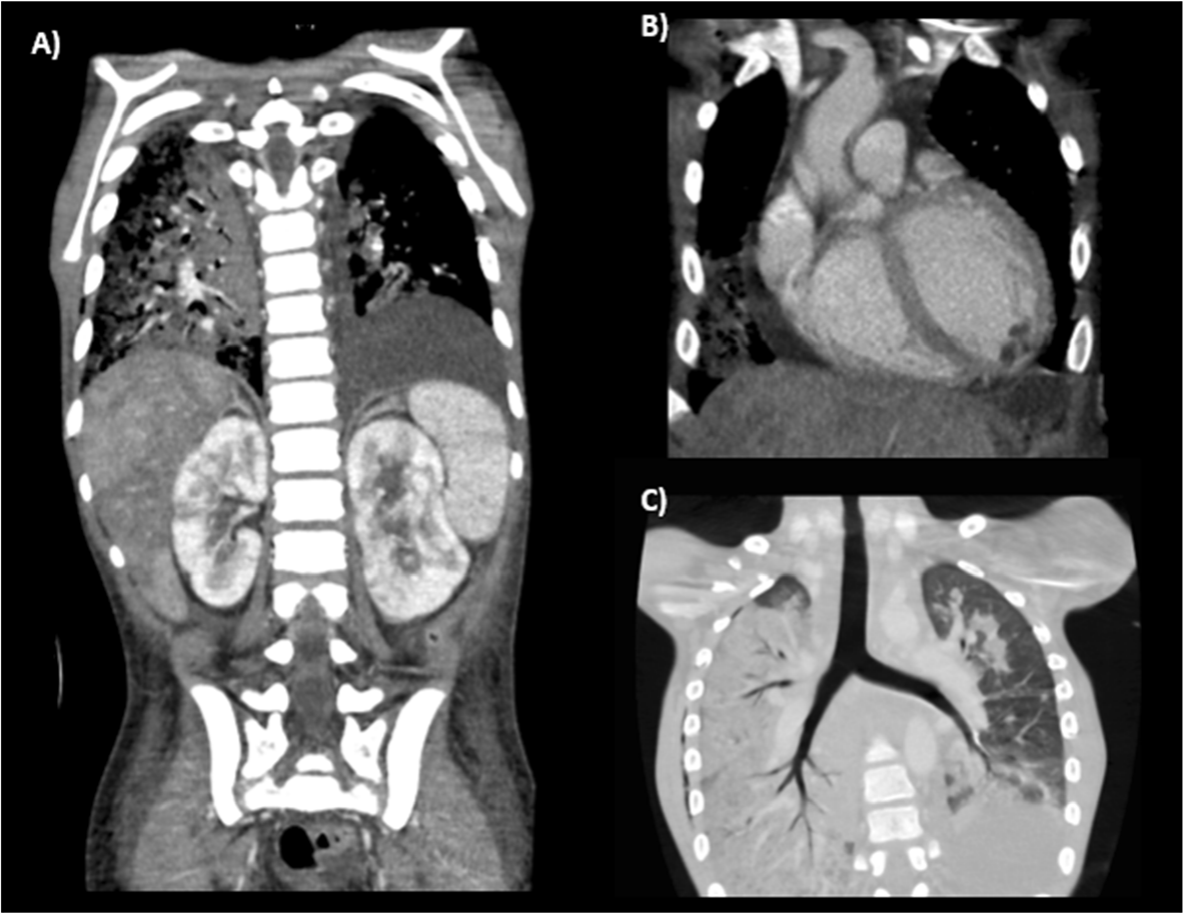
Coronal contrast-enhanced CT of the chest and abdomen. A and B) Soft tissue, and C) Lung algorithm. Images show extensive alveolar infiltrates with hepatisation of both lungs and moderate left pleural effusion. Diffuse cardiomegaly and hypovascular nodules located in septal and left apical ventricular subendocardium. Multiple ill-defined hypodense lesions predominantly involving the periphery of the liver, kidneys, and spleen. Enlarged mediastinal lymph nodes were also present (not shown)

Computed tomography scan showed extensive alveolar infiltrates in the lungs and multiple poor defined hypodense lesions in liver, spleen, and kidneys; adenopathy; and cardiomegaly with left ventricle subendocardial nodules. (Fig. 4) Tumour markers (neuron-specific enolase, alpha-fetoprotein, human chorionic gonadotropin), bone marrow biopsy, and serum amyloid A were normal. Pleural fluid, bronchial secretion, and skin biopsy revealed non-atypical lymphocytic infiltration.

**Fig. 5:**
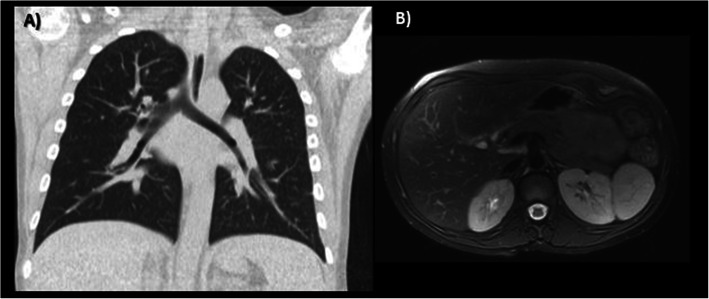
Complete resolution of pulmonary and abdominal viscera infiltrates is seen in A) Coronal non-enhanced lung CT, and B) Axial FS T2WI MRI

Invasive mechanical ventilation was started, and a pleura drainage tube was placed, together with broad antibiotic and antifungal coverage. After 4 days without significant improvement, Tocilizumab (8 mg/kg) and pulsed intravenous methylprednisolone (500 mg three consecutive days) were initiated. Improvement within a few days was impressive: mechanical ventilation could be stopped, inflammation markers and liver and kidney function normalized, heart function stabilized, achieving 50% of LVEF. Two weeks later, he was able to be discharged and has been clinically well and stable since then, only with administration of Tocilizumab every two weeks. A control CT scan done 10 weeks later showed complete resolution of previous abnormalities (Fig. 5).

## Discussion

To the best of our knowledge, this is the first case of HS reported in the literature presenting with acute cardiogenic shock; although two patients have been described with progressive heart failure during the course of the disease [14, 15]. Previous studies have shown that the spectrum of cardiac involvement in HS is widely diversified: mild pulmonary stenosis, pericardial effusion, pulmonary hypertension, ductus arteriosus with right to left shunt, atrial and ventricular septal defects, mitral valve prolapse, cardiomegaly, and myocardial hypertrophy [1, 16, 17]. The persistence of the left superior vena cava, as in our patient, has also been described previously [5].

It has been hypothesized that the expression of mutant hENT3 in the heart, affecting nucleoside transport activity, could interfere with normal cardiac activities and morphogenesis [4].

Our patient showed diffuse multiorgan infiltration in liver, spleen, lungs, and kidneys on a whole-body CT scan. (Fig. 4) Similar findings were described in another patient, although they were limited to bone marrow only and, as such, macrophage proliferation was postulated as the cause [10]. Extensive infiltration of multiple organs, possibly affecting the heart as well, as shown by the subendocardial nodules, has not been described previously and might represent severe disease activity leading to the acute life-threatening presentation. However, in our patient, bone marrow biopsy was normal, and another organ biopsy was not performed.

Two months after starting Tocilizumab, a control lung CT scan and abdominal MRI (Fig. 5) were normal without any signs of multiorgan infiltration. Therefore, the rapid improvement after initiation of treatment would be suggestive of a transient cell infiltrate as the underlying cause of the initial imaging findings.

The patient presented purpuric lesions from the 2nd to the 4th toes, suggesting a thrombotic or embolic aetiology in the context of his cardiomyopathy and severe autoinflammatory syndrome. As far as we know, digital ischemia has not been described in HS; although similar acral ischemic lesions have been described in severely ill patients during the COVID-19 outbreak [18]. Nonetheless, SARS-CoV-2 polymerase chain reaction (PCR) during the acute phase, and antibody detection by chromatographic immunoassay during the acute phase and after 6 weeks were negative.

As we mentioned before, we found the same mutation in her sister. This lack of phenotype-genotype correlation, even in siblings with identical mutations, had been already described in other case reports [3, 7, 12]. It highlights the importance of knowing the different diagnostic features of this disease to make an early-diagnosis and possibly preventing long-term complications.

hENT3 is critical for nucleotide synthesis by maintaining lysosome integrity. Thus, hENT3 deficiency affects apoptotic cell clearance and can increase macrophage colony-stimulating factor (MCSF) signalling. MCSF enhances macrophage and histiocyte proliferation but alters their function, with macrophage infiltration observed in multiple organs of animals with ENT3 deficiency [19, 20]. This contributes to elevated cytokine excretion and the immune response, resulting in skin sclerosis and hypertrichosis [11]. In this way, therapeutic benefit could be suggested for anti-cytokine biologicals. However, several of these treatments have not been effective, with various studies showing poor response to anti IL-1 or TNF-alpha treatment, despite high serum levels of IL-1 and TNF [3, 10, 13, 16, 21, 22]. Similarly, Mistry et al. reported a patient with no clinical response, but with c-reactive protein normalization after Tocilizumab treatment [23].

Our patient showed a very good response, in line with recent reports on successful treatment with intravenous Tocilizumab [3, 21].

Interestingly, interleukin-6 also plays an important role in cytokine release syndrome of COVID-19 [24]. Our patient had tested negative for COVID19. However, we cannot completely rule out COVID19 as an aggravating factor in this HS, as false negative PCRs and serologies have been reported [25].

## Conclusions

We report the most severe disease course produced by HS described so far in the literature. Our patient’s manifestations included uncommon, new complications such as acute heart failure with severe systolic dysfunction, multi-organ infiltration, and digital ischemia.

Most of the clinical symptoms of our patient could have been explained by SARS-CoV-2, demonstrating the importance of a detailed differential diagnosis to ensure optimal treatment.

Although the mechanism of autoinflammation of HS remains uncertain, the good response of our patient to Tocilizumab makes a case for the important role of IL-6 in this syndrome and for Tocilizumab as a first-line treatment, at least in severely affected patients.

## Data Availability

All data generated or analysed during this study are included in this published article.

## Abbreviations

SLC29A3: solute carrier family 29, member 3
hENT3: human equilibrative nucleoside transporter-3
PHID: insulin-dependent diabetes syndrome
SNHL: sensorineural hearing loss
IDDM: insulin-dependent diabetes mellitus
COVID-19: Coronavirus Disease-2019 pandemic
LVEF: left ventricular ejection fraction
PCR: polymerase chain reaction
MCSF: macrophage colony-stimulating factor
